# An Essential Role of Polymeric Adhesives in the Reinforcement of Acidified Paper Relics

**DOI:** 10.3390/polym14010207

**Published:** 2022-01-05

**Authors:** Jiaojiao Liu, Huiping Xing, Yajun Zhou, Xiaolian Chao, Yuhu Li, Daodao Hu

**Affiliations:** Engineering Research Center of Historical Cultural Heritage Conservation, Ministry of Education, School of Materials Science and Engineering, Shaanxi Normal University, Xi’an 710119, China; liujiaojiao@snnu.edu.cn (J.L.); xhp@snnu.edu.cn (H.X.); zhouyajun@snnu.edu.cn (Y.Z.); chaoxl@snnu.edu.cn (X.C.)

**Keywords:** polymeric adhesives, paper relics, back support, reinforcement and deacidification, conservation

## Abstract

Paper acidification causes paper relics to undergo embrittlement and decay, to form dregs, and even to break upon a single touch; therefore, reinforcement and deacidification treatments are essential steps for paper conservation and to retard the deterioration and prolong the life of objects. Polymeric adhesives play an essential role in reinforcement and deacidification treatments, although it is not well studied. In this work, the effect of polymeric adhesives on the conservation process and their protective effects on acidified paper relics were studied. Firstly, three polymeric adhesives, including wheat starch paste, polyvinyl butyral (PVB), and polyvinyl alcohol (PVA), were selected as research objects. Subsequently, their effects on four popular conservation methods were further discussed, including traditional mounting, hot-melt with silk net, alcohol-soluble cotton mesh, and water-soluble cotton mesh. Additionally, as an example, the reversibility and long-term durability of water-soluble adhesive PVA-217 were assessed. Using a computer measured and controlled folding endurance tester, pendulum tensile strength tester, tear tester, burst tester, FT-IR, video optical contact angle tester, and other instruments, the conservation application of water-soluble adhesives in paper relics was evaluated. This study provides a scientific basis and experimental data for the application of polymeric adhesives in the conservation of paper relics.

## 1. Introduction

Paper has been widely used as a popular information carrier to record and present history and culture in the area of cultural relics [1,2,3,4]. However, the current preservation situation of precious paper relics in modern times is not optimistic. Most of the paper relics published in this period, such as books, newspapers, and periodicals, have turned yellow and brittle, which may be because most of them are made of acid-based paper produced before the 1980s [5,6]. According to statistics [7], 80% of modern paper relics in large museums in China have acidification and yellowing, nearly 50% of them are damaged, and 30% are severely deteriorated, aging, and in urgent need of protection. Therefore, it is necessary to repair and protect these paper relics by reliable deacidification and reinforcement technologies.

Numerous deacidification methods have been studied in paper conservation; they are mainly classified into aqueous, non-aqueous, and gaseous deacidification methods. Calcium hydroxide, calcium bicarbonate, magnesium hydroxide, magnesium bicarbonate and magnesium oxide are the most commonly used deacidification reagents [8,9,10,11]. As paper acidification causes paper relics to undergo embrittlement and decay, to form dregs, and even to break upon a single touch, reinforcement treatment should be an essential step for these acidic paper relics [12,13,14]. Especially, for acidic paper relics with lost mechanical strength, a back support to enhance their mechanical strength is usually required. At present, several back supports have been successfully applied in these acidic paper relics, which mainly include Japanese tissue, Chinese Xuan paper, silk net, cotton mesh, and so on [15,16,17,18,19,20,21,22]. Japanese tissue is ideal for paper mending [18] due to its excellent properties in mechanical strength, weight, pH, color, fiber length, dimensional stability and resistance to aging. Mullock [19] proposed that Chinese handmade Xuan paper made from bast fiber are an alternative to Japanese tissue. Xuan paper is characterized by aging resistance, no discoloration, less moth damage, and long-term preservation, which can achieve a good reinforcement effect in paper relics. These two back supports were combined with wheat starch paste to reinforce fragile paper relics using a room-temperature mounting method. Subsequently, the National Library of the United Kingdom was the first to adopt a silk net with hot-melt adhesive to reinforce paper relics with double-sided writing. In our laboratory, cotton mesh has been proposed as a suitable back support to enhance the mechanical strength of acidic paper relics. In the beginning, cotton mesh combined with alcohol-soluble adhesive was first applied for paper conservation at room temperature [20]. The mechanical properties and durability performance of acidic paper samples were much improved after cotton mesh reinforcement. Recently, our research identified a simple method to replace an alcohol-soluble adhesive with a water-soluble adhesive that could achieve comparable reinforcement effects [21]. Therefore, polymer adhesives play an essential role in the conservation of paper relics. 

Polymer adhesives have been developed to combine different materials for a wide variety of application requirements in many other areas, including concrete structures [23], carbon nanotube sheets [24], wound closure [25], wood-to-wood bonding [26], 3D printed polymeric materials [27], and so on. The properties of polymeric adhesives were investigated through various characterization and measurements under normal load conditions [28] and extreme weathering conditions [29]. Previous research results indicated that different antimicrobials require different types of polymeric adhesives for effective coating [30], and a set of rational guidelines for the design of polymeric tissue adhesives was presented [31]. Grinstaff [25] reported how to design and synthesize polymeric hydrogel adhesives via physical and chemical methods to achieve excellent performance. Although some polymer adhesives were employed to bond back supports and paper relics, to the best of our knowledge, there has been no previous research on the effect of polymer adhesives on the paper conservation process or how to assess and select suitable adhesives in the field of paper relics. 

**Figure 1:**
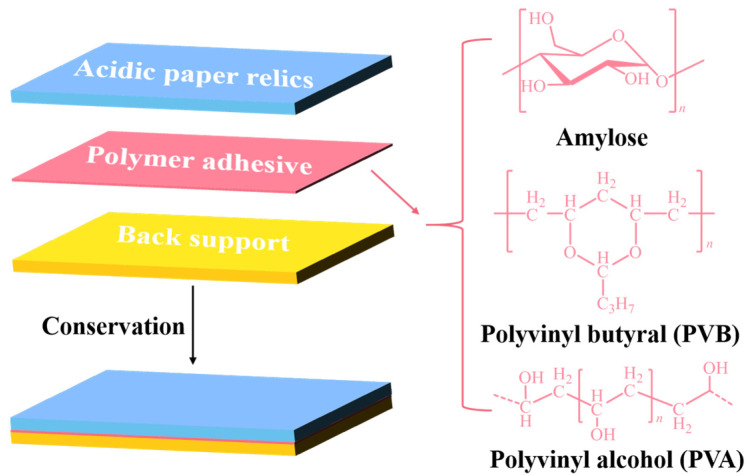
Schematic illustrations of paper conservation steps, and the molecular structures of polymer adhesives.

Based on the above discussion, three commercially popular polymer adhesives were selected as the research objects in this work, and their molecular structures are shown in Figure 1. These three polymer adhesives were picked out based on three aspects [32,33,34]. (1) Due to the precious nature of paper relics, polymer adhesives not only require excellent bonding characteristics for back supports and paper relics, but also need retreatment ability. (2) Polymer adhesives for rescuing a large number of paper relics need to be widely available and low-cost. (3) They should be non-toxic, odorless, colorless, and have good durability and mechanical properties. Firstly, the effect of these polymer adhesives on the paper conservation process has been comparatively studied in a traditional mounting method, hot-melt method with silk net, alcohol-soluble cotton mesh method, and water-soluble cotton mesh method. Secondly, the long-term durability of polymer adhesive and its effects on the mechanical properties of simulated newspaper samples were further evaluated. Finally, the application of water-soluble adhesive (PVA-217) as an example in the field of acidic paper relics was investigated. Overall, we expect to provide a scientific basis and experimental data for the future design and application of polymeric adhesives with excellent performance in the field of paper relics. At the same time, we also call on chemists and materials scientists to focus on the conservation of paper relics and apply our expertise to develop reliable restoration materials for the conservation of paper relics.

## 2. Experimental Section

### 2.1. Materials

Polyvinyl butyral (PVB, molecular weight: 40,000–70,000) was supplied by Macklin Reagent Co., Ltd., Shanghai, China. Wheat starch was available from Shing Fat Hong Enterprises Ltd., Hong Kong, China. Polyvinyl alcohol 217 (PVA-217, viscosity: 20.5–24.5 cps, alcoholysis degree: 87.0–89.0 mol%) was purchased from Tianjin Huachangyuan Chemical Trade Co., Ltd., Tianjin, China. MgO nanoparticles were purchased from Sichuan Ruili Cultural Relic Protection Technology Co., Ltd., Sichuan, China. Saturated magnesium carbonate hydroxide aqueous solution was reacted with CO_2_ for 24 h to prepare Mg(HCO_3_)_2_ solution. A single cotton filament with a diameter of 80–90 μm was selected and woven on a netting machine to form a square grid with the side length of 1.5 mm. Then, alcohol-soluble adhesive (PVB) or water-soluble adhesive (PVA-217) was sprayed onto the grid using a spray gun (pressure at 0.5~0.8 MPa) and dried for 24 h at room temperature. The spray gun head was 3 cm from the grid, and the thickness of the adhesive was 15~20 μm. Finally, a cut was made using scissors along the edge of the netting frame to obtain the cotton mesh from the netting frame. The silk net was provided by Nanjing Museum, Nanjing, China. The Japanese tissue was purchased from Beijing Meida Technology Co., Ltd., Beijing, China.

### 2.2. Sample Preparation

(1) Simulated newspaper samples: Modern newspapers were subjected to three processes to accelerate aging and prepare a sufficient number of simulated samples. First, 8 wt% gelatin–alum water was dropped from a pipette onto the surface of simulated newspaper samples; then it was brushed with a soft wool brush on the samples to adjust their pH before aging. After natural drying for 48 h, the average pH of the samples was measured to be between 4.5 and 5.5 using cold water extraction method. Then, the accelerate aging processes were performed.
(2) PVA-217 films: The 5 wt% PVA-217 was added into distilled water to prepare a transparent solution. This solution was injected into a polytetrafluoryl grinding tool, and a thin and uniform PVA-217 film was prepared by drying the solution at room temperature for 72 h.

### 2.3. Accelerated Aging Process

Three general methods were employed for the accelerated aging [35]. In this work, these accelerated aging methods were carried out on the tested samples, including a sufficient number of modern newspapers, PVA-217 films, simulated newspaper samples before and after restoration, and one old newspaper from the Republic of China before and after restoration. Ten parallel samples of PVA-217 films were used for each aging time. Each sample of PVA-217 film was aged for 3, 5, and 7 days. The first method was dry heat aging, in which the tested samples were placed in a blast drying aging box at 105 °C. The second method was hygrothermal aging, in which the tested samples were placed in a constant damp heat aging test box at a temperature of 80 °C and a relative humidity of 65%. The third method was ultraviolet light aging, in which an ultraviolet weather-resistant test box was used to age the test samples; the temperature of the aging box was set at 25 °C, the power of the ultraviolet lamp was 60 W, and the vertical distance between the sample and the ultraviolet lamp was 5 cm.

### 2.4. Characterization and Measurements

#### 2.4.1. Chromatic Aberration Test

The spectrophotometer (X-RiteVS-450, ASHLEY Instruments Inc., Greensburg, IN, USA) was applied to test the chromatic aberration ∆E* of the PVA-217 films before and after aging for 3 days, 5 days, and 7 days. Each sample was measured 5 times and the average value was obtained.

#### 2.4.2. Mechanical Strength Test

According to the direction of fiber arrangement in the papermaking process, paper can be divided into vertical and parallel directions. As the mechanical properties of paper in the vertical and parallel directions are very different, the simulated paper samples were divided into four groups before and after aging: untreated parallel, untreated vertical, treated parallel, and treated vertical samples. For each group, sufficient parallel samples were prepared, each sample was measured once, and finally ten valid data were used to obtain the average value. If the fracture is happened within 10 mm of the fixture, the tested data will be invalid. Mechanical strength tests mainly included tensile strength, folding endurance, tear and burst resistance tests. In order to control the variables, in the experiment, the simulated newspaper samples were cut from the vertical and parallel directions to obtain a sufficient number of test samples. Importantly, it is necessary to ensure the integrity of the test samples without any damage or disease, so as to ensure the authenticity of the data. The test details were as follows:

(1) Tensile strength: The tested samples had a length of 250 mm and a width of 15 mm. Using the pendulum tensile strength tester (J-KZ1000, Changjiang Paper Instrument Co., Ltd., Sichuan, China), the tensile strength of test samples was measured at a speed of 5 mm/min according to Standardization Administration of the People′s Republic of China (GB/T453-2002).
(2) Folding endurance: The tested samples had a length of 250 mm and a width of 15 mm. Using a computer measured and controlled folding endurance tester (DC-MIT135B, Changjiang Paper Instrument Co., Ltd., Sichuan, China), the folding endurance of test samples was measured according to Standardization Administration of the People′s Republic of China (GB/T2679.5-1995). The applied force was 4.9 N.
(3) Tearing resistance: The test samples had a length of 75 mm and a width of 63 mm. Using a computer measured and controlled tear tester (DC-SLY13K, Changjiang Paper Instrument Co., Ltd., Sichuan, China), the tearing strength of test samples was measured according to Standardization Administration of the People′s Republic of China (GB/T455-2002).
(4) Bursting strength: The test samples had a length of 70 mm and a width of 70 mm. Using a computer measured and controlled burst tester (DC-NPY1200, Changjiang Paper Instrument Co., Ltd., Sichuan, China), the bursting strength of test samples was measured according to Standardization Administration of the People′s Republic of China (GB/T450-2002).

#### 2.4.3. Water Contact Angle Test

To study the hydrophilic and hydrophobic properties of acidified, decayed newspapers and ancient books in the Republic of China before and after restoration, a video optical contact angle meter (OCA20, Dataphysics, Stuttgart, Germany) was employed to measure their static contact angles by sessile drop method [36,37,38,39,40,41]. The test details were as follows:

The measurement was performed at room temperature and in an air atmosphere. It should be noted that the needle tip cannot touch the tested sample during the test. The test liquid was deionized water and the injection speed was 2 μL/s. Each sample was measured in parallel at 5 different places; the contact angle value was their average value. The distance of test sample was about 1 cm away from the needle tip. A droplet with constant volume of about 2 μL was placed using a microsyringe onto the test sample, and then the static contact angle measurement was started after stabilizing the water droplets on the surface of test sample for about 1 s. An image of the drop was acquired by the camera and processed by the instrument software to determine the value of the contact angle with the method of tangent fitting. The contact angle was obtained without correction for evaporation effects.

#### 2.4.4. FT-IR Spectroscopy Test

Some fragments from a severely acidified and decayed newspaper from the Republic of China were collected as the test samples. One part of these samples was reinforced with cotton mesh and water-soluble adhesive PVA-217, while the other part was used as a blank comparison. The unreinforced samples were referred to the samples as blank comparisons. Fourier transform infrared spectrometer (FT-IR) was used to test reinforced samples and unreinforced samples. The wave number range was from 500 to 4000 cm^−1^ and the resolution was 4 cm^−1^. Infrared spectrometry was performed on fiber samples using the potassium bromide tablet method. To reduce the effect of KBr on FT-IR spectra, sample spectra had the background contributions removed. FT-IR tests were performed on the samples before and after dry heat aging, hygrothermal aging, and UV aging. The structural changes in the paper cellulose before and after restoration and before and after aging were analyzed. 

## 3. Results and Discussion

### 3.1. Effect of Polymeric Adhesive on Conservation Process

Based on the selected polymer adhesives in Figure 1, four popular conservation methods have been successfully applied for paper relics, including traditional mounting, hot-melt with silk net, alcohol-soluble cotton mesh, and water-soluble cotton mesh. Based on references [15,20,21,22] and restoration experience, the corresponding conservation workflows of these four conservation methods presented in Table 1 were carried out by ourselves to achieve a good restoration effect. After a comparative study of their conservation workflows, it is clear that the polymeric adhesive plays an essential role in the conservation process. In the experiment, the hot-melt adhesive required high-temperature ironing to adhere the silk net and paper relics, while other conservation methods only needed room temperature. The alcohol-soluble cotton mesh method employed large amounts of ethanol as a solvent during the conservation process, which may be a safety risk and harm operator health. The experimental phenomenon showed that the adhesive PVB was prone to agglomeration in an alkaline environment when soaked in ethanol. Hence, the alcohol-soluble cotton mesh method must apply reinforcement treatment before deacidification to ensure a good conservation effect. If the water-soluble adhesive is used to combine the back support and paper relics, deacidification is usually carried out before reinforcement treatment, which is attributable to the effect of aqueous deacidification reagents on the bonding ability of the water-soluble adhesive.

### 3.2. Long-Term Durability of Polymeric Adhesive

**Figure 2:**
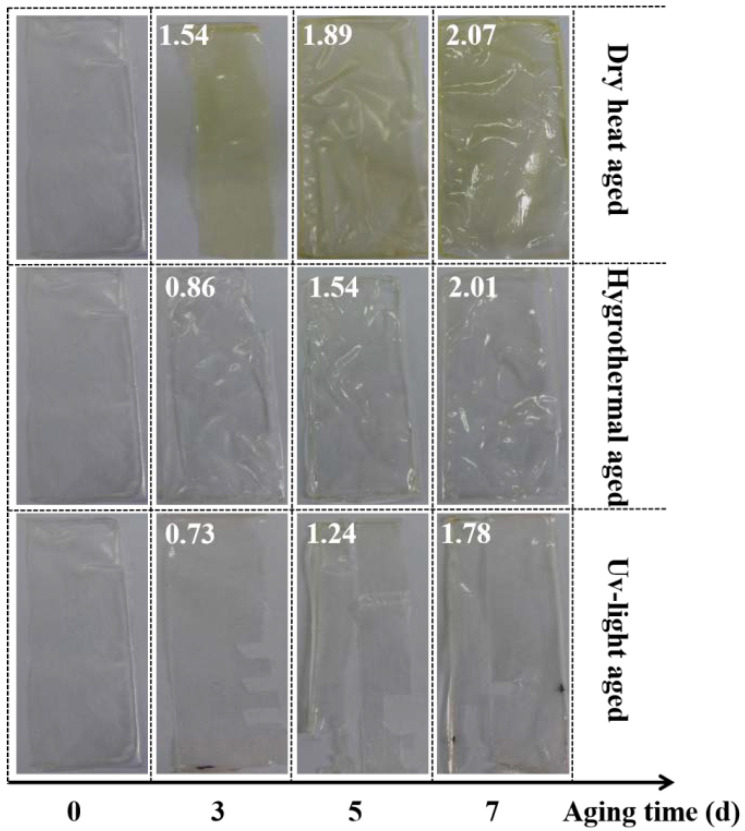
Effect pictures of PVA-217 films before and after aging several days by using three aging methods. The chromatic aberration data (white numbers) were listed in the effect pictures.

Water-soluble adhesives are preferred for the application of the large-scale restoration of paper relics. Wheat starch pastes tend to attract borers and nourish mildew, disadvantaging the long-term durability. In this study, dry heat, hygrothermal and UV-light accelerated aging methods were utilized to evaluate the durability of the water-soluble adhesive PVA-217. From the chromatic aberration data of the PVA-217 films combined with the intuitive diagram in Figure 2, the hygrothermal aging and UV aging had slight effects on the color of PVA-217. The dry heat aging caused relatively prominent color difference of the PVA-217 films. It is speculated that the yellowing resulted from the thermal oxidative aging of PVA-217. As PVA-217 undergoes thermal oxidation reaction in the dry-heat aging box at 105 °C, some hydroxyl groups were oxidized to carbonyl groups. As the aging time was prolonged, the content of the carbonyl group increased and the film color changed gradually from colorless and transparent to light yellow, indicating that the adhesive was more sensitive to the dry heat aging among the three accelerated simulated aging conditions. It is recommended to avoid an extreme high-temperature environment for the storage of paper relics after reinforcement with a water-soluble cotton mesh.

### 3.3. Effect of Polymeric Adhesive on Mechanical Properties of Simulated Samples

**Figure 3:**
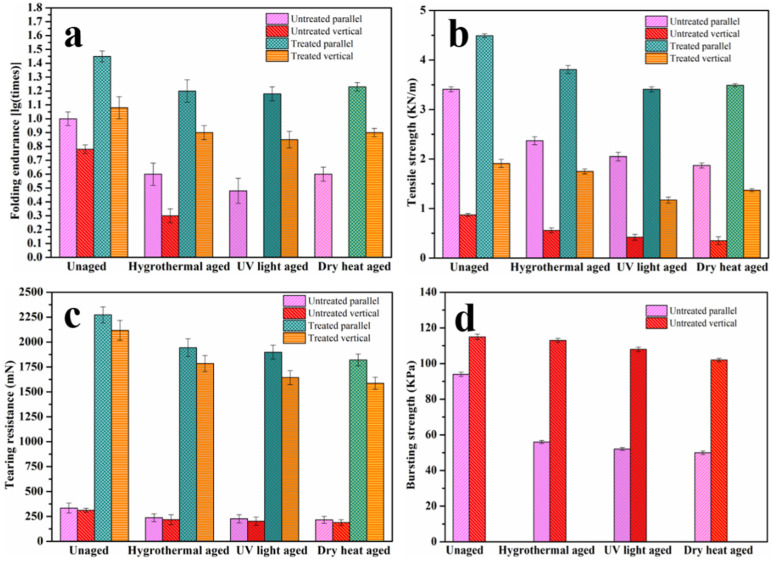
Test results of the mechanical properties of simulated samples before and after aging and restoration were as follows: folding endurance (**a**); tensile strength (**b**); tearing strength (**c**); bursting strength (**d**).

Mechanical strength tests are destructive tests; therefore, some simulated newspaper samples were employed to study the effect of the polymeric adhesive on mechanical properties. The folding endurance refers to the number of times that the paper can be folded by 180 degrees under a certain tension. When the shear force used in the tensile strength test was increased to exceed the shear strength of the paper fiber, the paper fiber will be broken. The tearing resistance test needs to tear the paper fiber by a blade in the tear tester. A pressure is gradually applied to the paper through an elastic film in the burst tester; when the test sample is broken, the measured maximum pressure is the bursting strength. As shown in the overall trend in the test results in Figure 3, the restoration via the cotton mesh and water-soluble adhesive PVA-217 can enhance various mechanical strengths, including the folding endurance, tensile strength, tearing resistance, and bursting strength of the newspaper samples. Furthermore, the simulated newsprint paper samples before and after restoration were subjected to hygrothermal aging, UV light aging, and dry heat aging to accelerate the aging process. It was found that the mechanical properties of the simulated original paper samples decreased significantly after aging, while those of the samples after restoration decreased slightly, implying a higher retention rate. The reasons are that the added pure cotton mesh is composed of pure cotton fiber with 100% cellulose content and that lignin does not affect the rate of aging of the paper [42]. The results showed that water-soluble adhesive PVA-217 combined with cotton mesh and fragile paper relics to meet the current requirements for the mechanical properties after reinforcement and restoration.

### 3.4. Conservation Application of Polymeric Adhesive in Paper Relics

#### 3.4.1. Reversibility of Polymeric Adhesives

**Figure 4:**
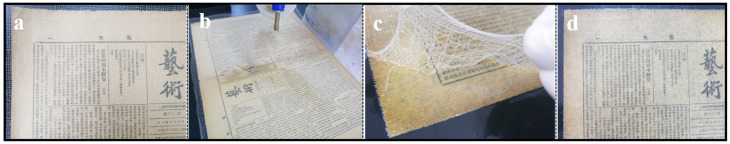
Reversibility process: before (**a**) and after (**d**) removing reinforcement material; spraying of ultra-pure water (**b**); of removal of cotton mesh (**c**).

It is necessary to take reversibility into full consideration for restoration methods for paper relics, but this requirement increases the difficulty of choosing appropriate polymer adhesives. The reversibility of paper documents refers to the concept that after the reinforcement, when the development of science and technology provides better protective materials, the original reinforcement material can be completely removed or removed as much as possible after a few years. Therefore, the paper document reinforcement technology and polymeric adhesive require good reprocessability after reinforcement. In this study, PVA-217 is employed as an example of water-soluble adhesives. The water-soluble cotton mesh method was employed to reinforce the newspaper from the Republic of China. The reprocessability of polymeric adhesives before and after treatment is shown in Figure 4. By moistening the reinforced sample (Figure 4a) with an appropriate amount of distilled water (Figure 4b), the cotton mesh can be separated from the paper after a few minutes of moistening (Figure 4c). The paper is not damaged during the removal and separation process (Figure 4d). However, it makes no sense to consider the reversibility of a treatment if the original is already so brittle that it cannot withstand immersion. 

#### 3.4.2. Effect of Paper Hydrophilicity and Hydrophobicity

**Figure 5:**
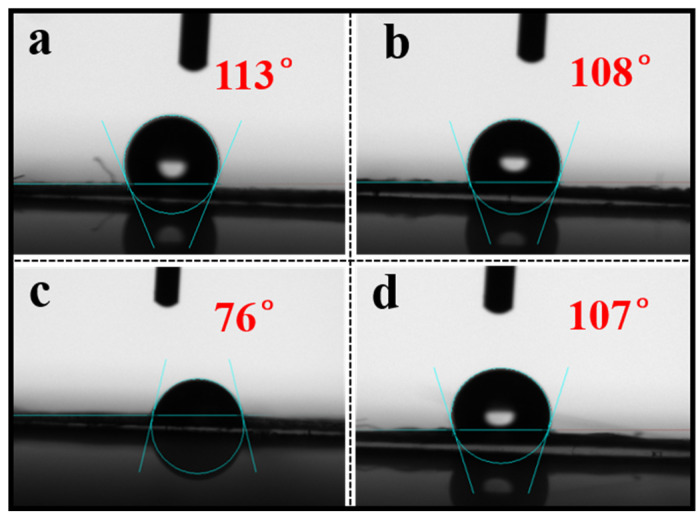
The contact angle photos of various paper relics: before (**a**) and after (**b**) restoration of the newspaper; before (**c**) and after (**d**) restoration of the books.

Two different types of paper relics (ancient acidified, decayed newspaper, and book) were used in the water contact angle tests and the results are shown in Figure 5. From Figure 5a,b, the contact angle of the original newspaper from the Republic of China was 113°, indicating that the paper surface was hydrophobic. The video contact angle of the paper reinforced with cotton mesh was 108°, which was slightly lower than that of the original paper. The decrease shows that the conservation by using the cotton mesh and water-soluble adhesive PVA-217 had little effect on the original hydrophobic properties of the paper. For the books from the Republic of China (Figure 5c,d), the contact angle was 76° before the restoration, demonstrating a hydrophilic state. After the restoration, the contact angle was amplified to 107°. These results indicate that the contact angle of the paper after the restoration increased. This conferred certain hydrophobic properties to the paper and slowed down the moisture absorption of the paper cellulose and reduce the excessive contact of the cellulose molecules with the moisture in the air under high humidity, effectively alleviating the hydrolysis reaction of the cellulose molecules [43,44]. In general, the cotton mesh as a reinforcing layer had a slight effect on the hydrophilicity and hydrophobicity of the hydrophobic paper. However, for the hydrophilic paper, the restoration enhanced its hydrophobic performance, which is conducive for the long-term preservation of the paper archives and documents [45].

#### 3.4.3. Effect of Long-Term Durability

Follow-up observations could effectively evaluate the long-term durability of paper relics after restoration. In recent years, we have used the above-mentioned water-soluble cotton mesh method to treat more than 1200 pieces of the Republic of China newspaper and paper archives of books and historical materials in the collections of Xi’an Eighth Route Army Office, Jinan Municipal Archives, Dali County Archives, and others, by deacidifying and reinforcing with cotton mesh and water-soluble adhesive PVA-217. The original newspaper and paper archives had varying degrees of acidification, aging, damage, and decay. The follow-up observations suggest that these paper relics after restoration no longer continued to suffer from deterioration, acidification, embrittlement, slag drop and so on, and remained stable.

**Figure 6:**
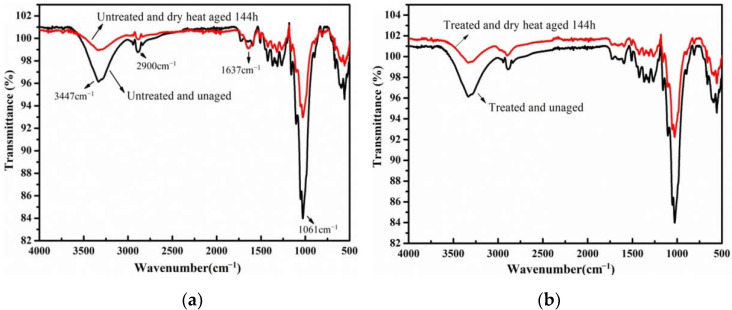
The IR spectra of paper-based documents before and after dry-heat aging: untreated (**a**) and treated (**b**).

**Figure 7:**
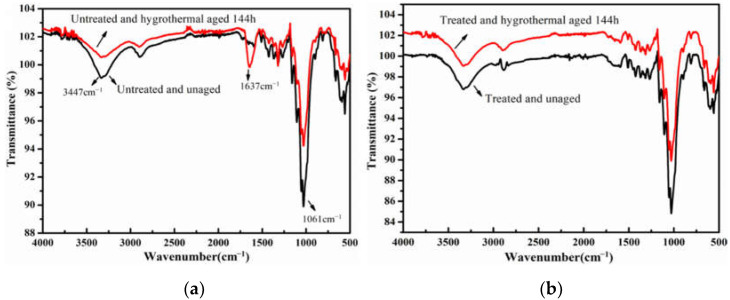
The IR spectra of paper-based documents before and after hygrothermal aging: untreated (**a**) and treated (**b**).

**Figure 8:**
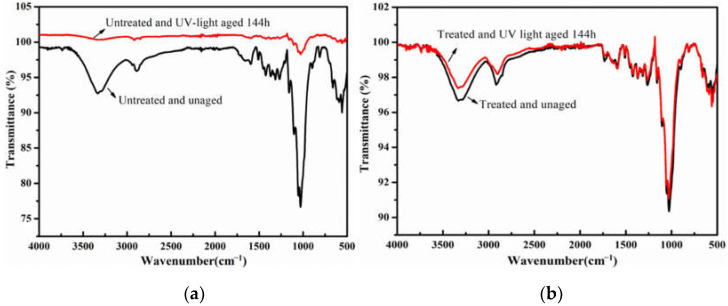
The IR spectra of paper-based documents before and after UV aging: untreated (**a**) and treated (**b**).

Meanwhile, three accelerated aging methods combined with FTIR technology were employed to evaluate the effect of long-term durability after the water-soluble cotton mesh restoration. The FTIR test results are shown in Figure 6, Figure 7 and Figure 8. As per infrared (IR) test results in Figure 6 and Figure 7, and from the comparison of the IR test analysis of the old newsprint after dry heat aging and hygrothermal aging, the hydroxyl peak of the original paper sample at 3384 cm^−1^ was weakened after dry heat and hygrothermal aging. The vibration peak of the carbonyl group (C=O) at 1673 cm^−1^ was clearly enhanced, and the C-O group peak at 1061 cm^−1^ was weakened. Hence, in the original newsprint, the hydroxyl groups of cellulose were oxidized to carbonyl groups under the accelerated aging of dry heat and hygrothermal conditions. The IR test analysis on the newsprint after reinforcement shows that there were three main characteristic peaks of paper cellulose, namely 2900 cm^−1^, 3400 cm^−1^, and 1040 cm^−1^, which remain pronounced after reinforcement with pure cotton mesh. No new characteristic peak was generated. The reinforcement did not bring any new groups to the paper fibers nor affected the main functional groups in the paper. This is mainly because pure cotton fiber and paper are made of plant fibers, and the structure of the adhesive polymer is simple, primarily containing C-C, C-H, and -OH groups. The absorption peaks formed by these groups were covered by the main characteristic peaks of the paper fibers. 

As per the IR test results of samples subjected to UV light (Figure 8), the main functional groups of cellulose in the original paper sample were significantly weakened or disappeared after UV aging, which indicated that the UV aging has a significant degradation effect on the paper fibers. The peak positions of the cellulose in the reinforced paper after UV aging were basically the same as those before aging. From the comprehensive comparison of the IR test results for the three aging processes, the reinforcing effect of the pure cotton mesh delayed the aging and decreased the degradation rate of the paper. In addition, among the three simulated accelerated aging systems, UV light aging had the greatest impact on the properties of newsprint paper. The accelerated aging with a duration of 144 h can degrade cellulose molecules in paper to a relatively greater extent.

## 4. Conclusions

To evaluate the durability, removability, and feasibility of polymeric adhesives in protecting acidic paper relics, three polymeric adhesives, including wheat starch paste, polyvinyl butyral, and polyvinyl alcohol, were selected to study their influences on the paper conservation process and protective effects. After comparative evaluation of the conservation workflows of three polymeric adhesives, it is concluded that polymeric adhesives play an essential role in the conservation process, especially with regard to the operation temperature, treatment sequence of reinforcement and deacidification, and process complexity. Water-soluble adhesives have the advantages of convenient operation, safety, and environmental protection, which are preferred for application in the large-scale restoration of paper relics. PVA-217 is as an example of water-soluble adhesives to be assessed, showing not only long-term durability, but was also in line with the reprocessing principle. Water-soluble adhesive PVA-217 combined with cotton mesh and fragile paper relics to meet the current requirements for the mechanical properties after reinforcement and restoration. After the hydrophilic paper was reinforced, the paper displayed a certain hydrophobicity that could slow down the moisture absorption by the cellulose in the paper. Follow-up observations and IR spectroscopy analysis indicated that the conservation application of polymeric adhesive helped to delay the aging and degradation rate of cellulose molecules in the paper. Although there are few reports on the application of polymeric adhesives in paper relics, it is believed that the design and synthesis of novel polymeric adhesives would provide additional support to this endeavor in paper relics. Next-generation polymeric adhesives in acidic paper relics should be developed with a holistic understanding of the physicochemical properties of polymeric adhesive, actual paper conservation processes, and requirements for specific applications.

## Figures and Tables

**Table 1:** Comparison of four popular conservation methods.

Conservation Method	Conservation Workflow
Traditional mounting method	Flatten the paper with distilled water; Deacidify with MgO nanoparticles; Brush the wheat starch paste (water-soluble adhesive) on the paper; Line the paper with Japanese tissue; Absorb excess water and adhesive; Dry under normal atmospheric conditions.
Hot-melt method with silk net	Flatten the paper with distilled water; Deacidify with MgO nanoparticles; Dry under normal atmospheric conditions; Spray the hot-melt adhesive (PVB) evenly on the silk net; Adhere the above silk net and the paper by ironing at high temperature.
Alcohol-soluble cotton mesh method	Spray the alcohol-soluble adhesive (PVB) evenly on cotton mesh; Reinforce the paper with above cotton mesh and ethanol; Dry under normal atmospheric condition; Deacidify with Mg(HCO_3_)_2_ aqueous solution; Dry under normal atmospheric conditions.
Water-soluble cotton mesh method	Flatten the paper with distilled water; Deacidify with Mg(HCO_3_)_2_ aqueous solution; Spray the water-soluble adhesive (PVA-217) evenly on cotton mesh; Reinforce the paper with the above cotton mesh and water; Dry under normal atmospheric conditions.

## Data Availability

The data presented in this study are available on request from the corresponding author.
